# Supplementary material for: Metamorphism in TDP-43 prion-like domain determines chaperone recognition
Source: Nat Commun. 2023 Jan 28;14:466. doi: 10.1038/s41467-023-36023-z (PMC9884275; doi:10.1038/s41467-023-36023-z)
Supplement: Supplementary file 1 — Supplementary Information [file 41467_2023_36023_MOESM1_ESM.pdf]

Metamorphism in TDP-43 prion-like domain determines chaperone recognition

Jaime Carrasco, Rosa Antón, Alejandro Valbuena, David Pantoja-Uceda, Mayur Mukhi, Rubén Hervás, Douglas V. Laurents, María Gasset, Javier Oroz

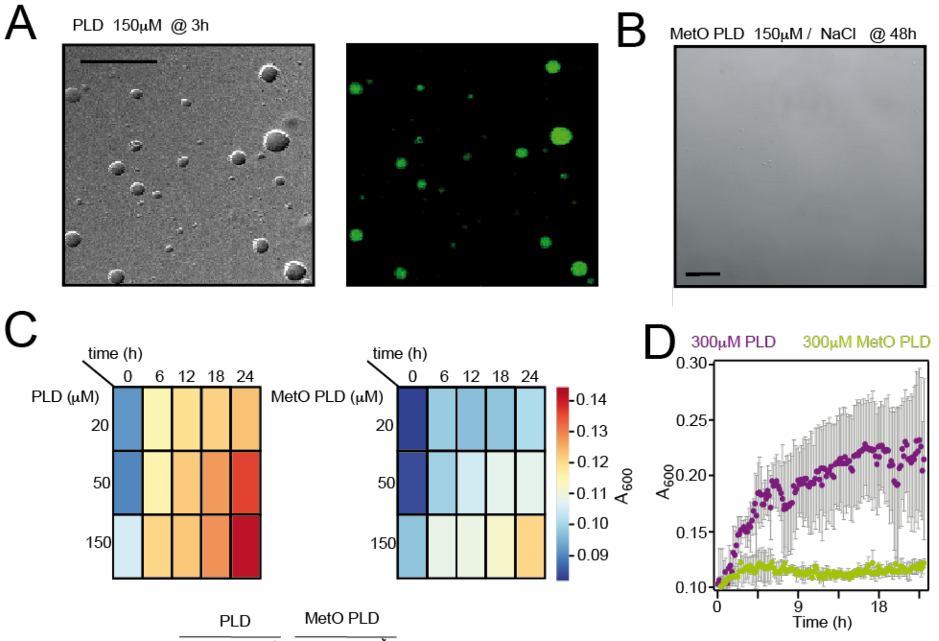

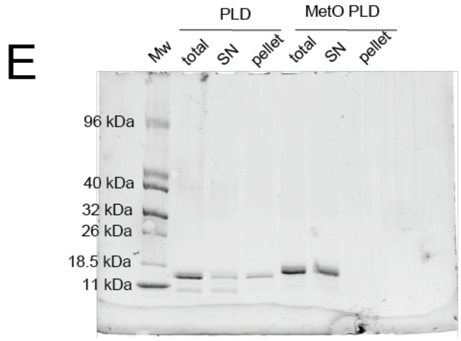

F

	soluble fraction		
	(μM)	25 °C, agitation (turbidity cond.)	15 °C, no agitation (NMR cond.)
PLD	20	95 +/- 5%	-
	150	65 +/- 5%	-
	200	-	35 +/- 3%
MetO PLD	20	100%	-
	150	100%	-
	200	-	100%

Supplementary Figure 1. Methionine sulfoxidation abrogates TDP-43´s PLD phase separation. A) DIC (left) and fluorescence microscopy (right) images for 150 μM PLD incubated for 3h at 25 °C. Images were acquired on the same sample field. Sample contained 150 mM KCl (turbidity buffer). **B)** DIC microscopy image for 150 μM MetO PLD incubated for 48h at 25 °C showing the absence of condensates. Sample contained 150 mM NaCl to promote LLPS (**Figure 1B**). **C)** Heat map representation for PLD (left) and MetO PLD (right) concentration-dependent LLPS at 25 °C, as assessed by turbidity measurements. Samples contained 150 mM KCl, and measurements were replicated. The color scale is indicated on the right. **D)** Turbidity measurements showing LLPS for the PLD at 25 °C. 300 μM protein concentration and 10 mM KCl were used, which correspond to the NMR conditions used in (**Figures 1E-F, Supplementary Figures 3-7**). **E)** SDS-PAGE of 200 μM samples incubated at 15 °C for 48 hours. Samples contained 10 mM KCl (NMR buffer). While no MetO PLD was observed in the pellet fraction, only 35% of the PLD remained in the soluble fraction (SN). PLD is 13.8 kDa. **F)** De-mixing of the PLD as quantified by gel band densitometry in turbidity (150 mM KCl, corresponding to panel **C**) and NMR (10 mM KCl, corresponding to panel **E**) conditions after 48 h of incubation. No band was observed in the insoluble fractions for any MetO PLD samples. Scale bars in (**A**) and (**B**) correspond to 25 μm. Related to **Figures 1B-D.**

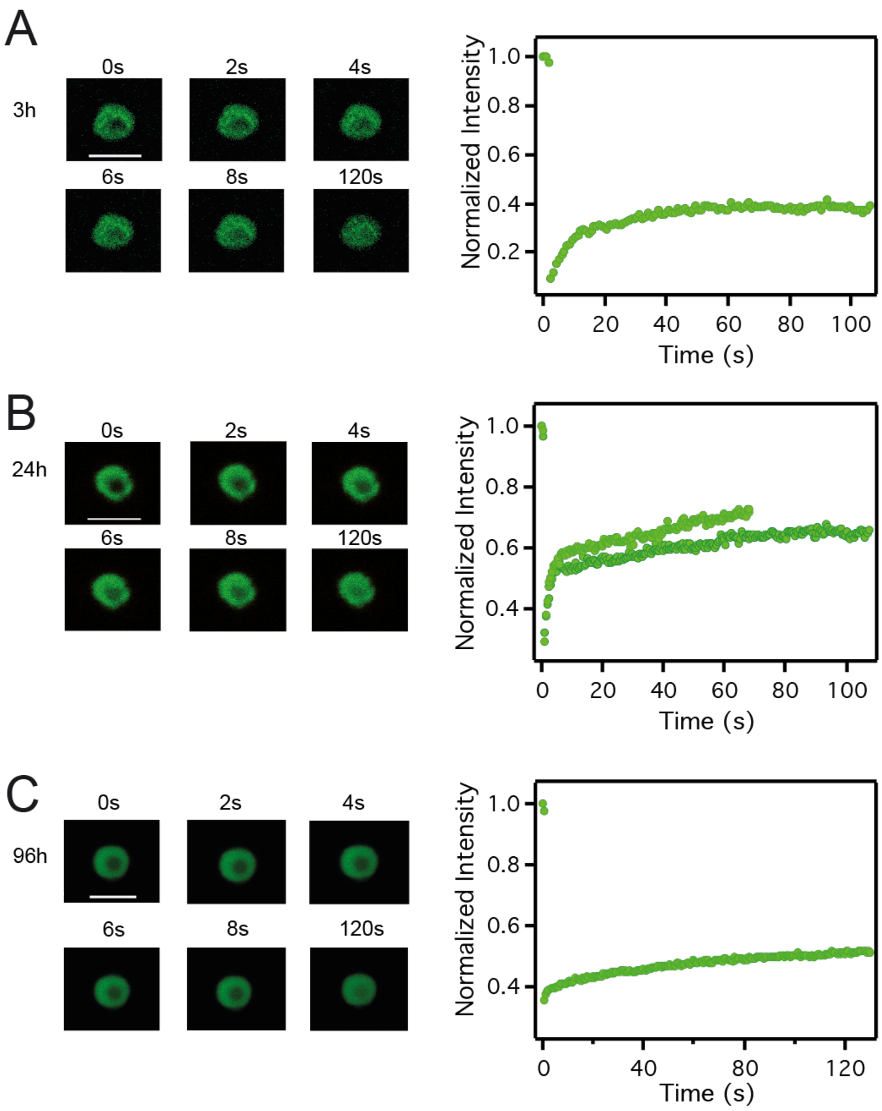

Supplementary Figure 2. TDP-43´s PLD phase separates into fluid droplets. A-C) Fluorescence recovery after photobleaching (FRAP) experiments for representative condensates formed by the PLD after 3 (**A**), 24 (**B**) and 96 hours (**C**) of incubation at 25 °C in turbidity conditions. The condensates remained fluid even after long incubation times, which largely cover the NMR measurement time required for the assignments and relaxation data reported in this study. 45 - 50 % intensity recovery after 60 s was determined for the upper condensates (**A, B**), while the condensate at the bottom (**C**) displayed a 25 % intensity recovery after 120 s. Two consecutive FRAP experiments were acquired on the condensate in (**B**) demonstrating the fluidity of the PLD condensates. Scale bars represent 5 μm. Error bars (not included) are below 0.25 on the normalized scale. Related to **Figure 1C.**

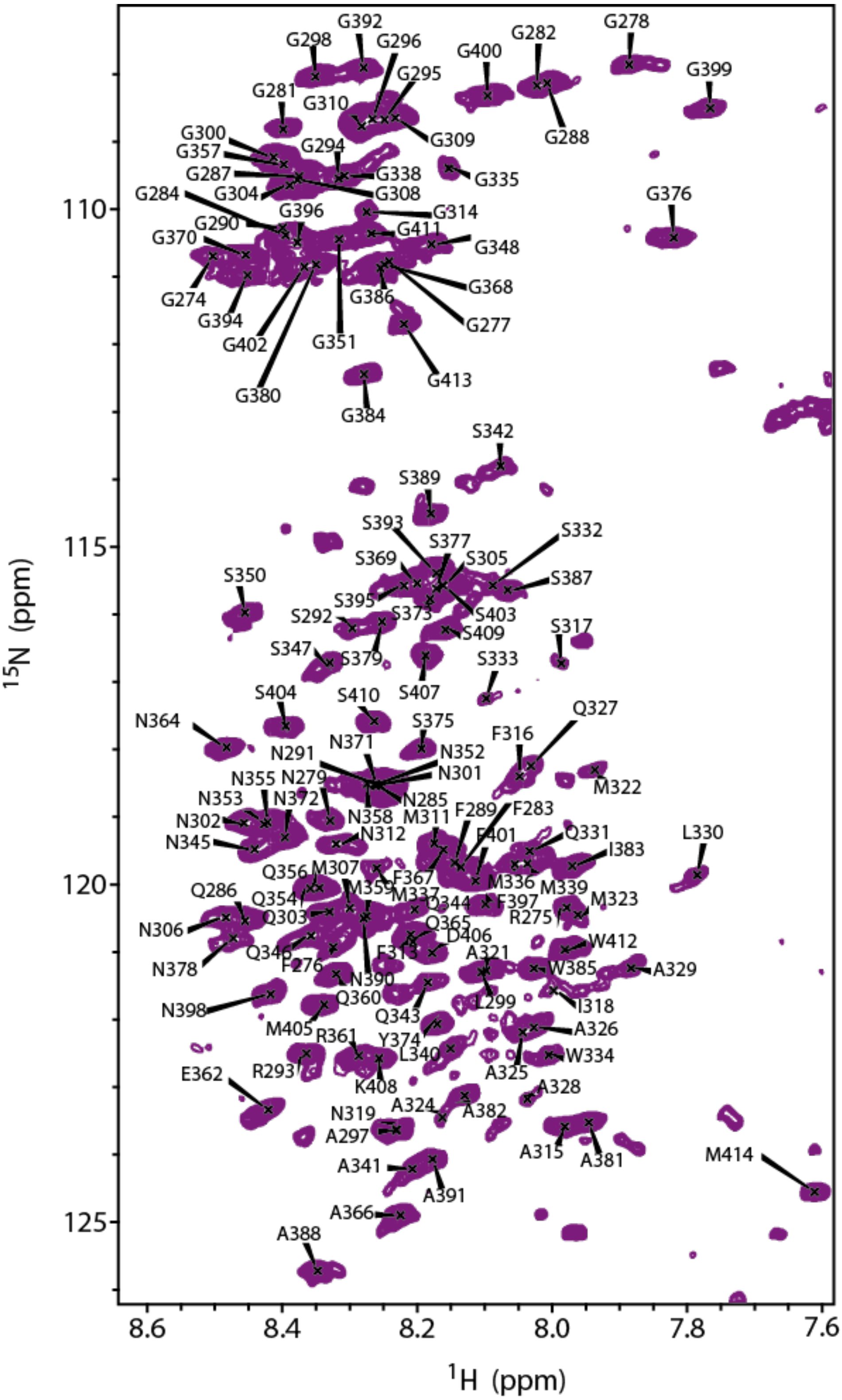

Supplementary Figure 3. Assigned ^{15}N-HSQC spectrum of de-mixed PLD. The spectrum corresponds to 300 μM PLD. Related to **Figure 1E.**

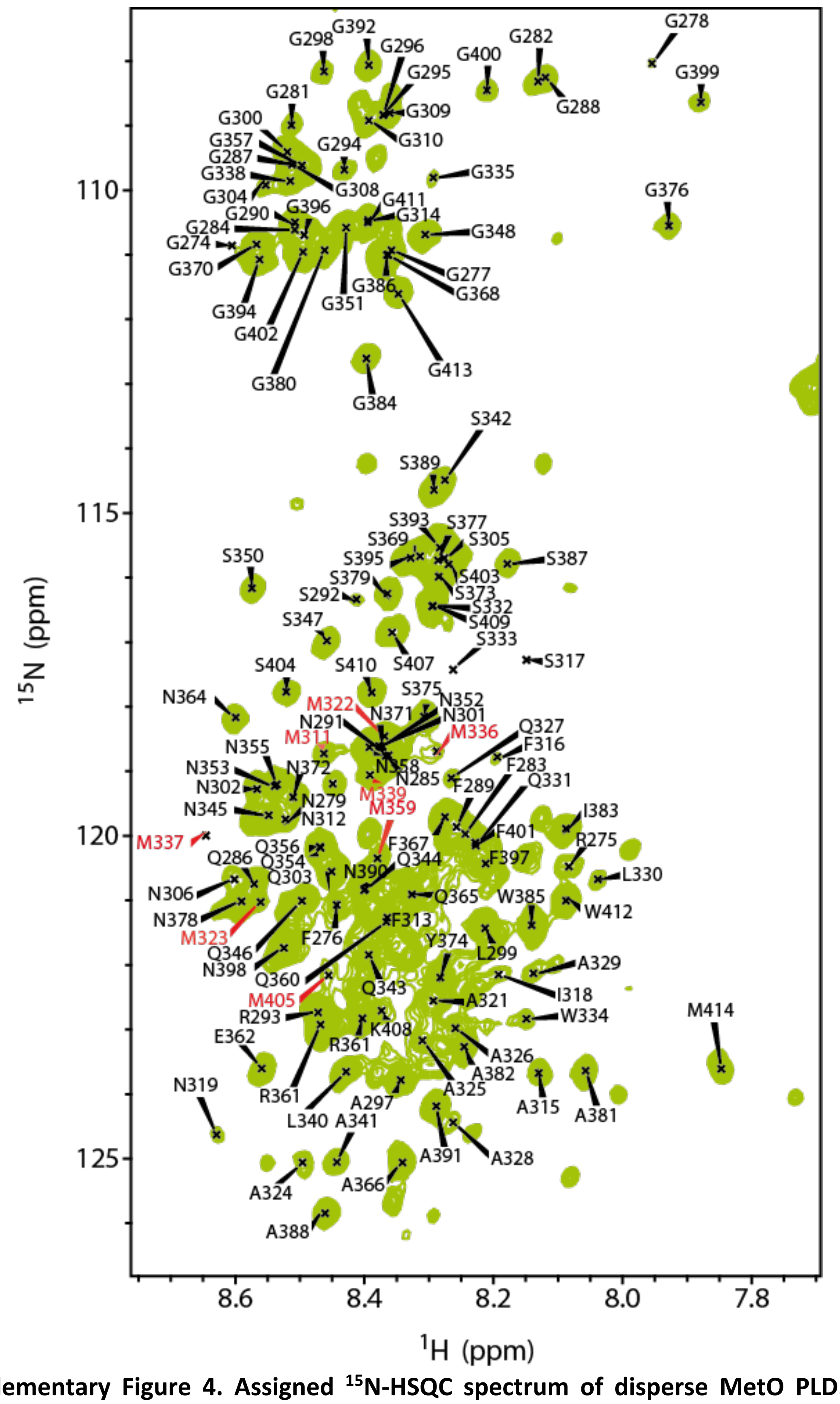

Supplementary Figure 4. Assigned ^{15}N-HSQC spectrum of disperse MetO PLD. The spectrum corresponds to 300 μM MetO PLD. Sulfoxidized methionines are labeled in red. Related to **Figure 1E.**

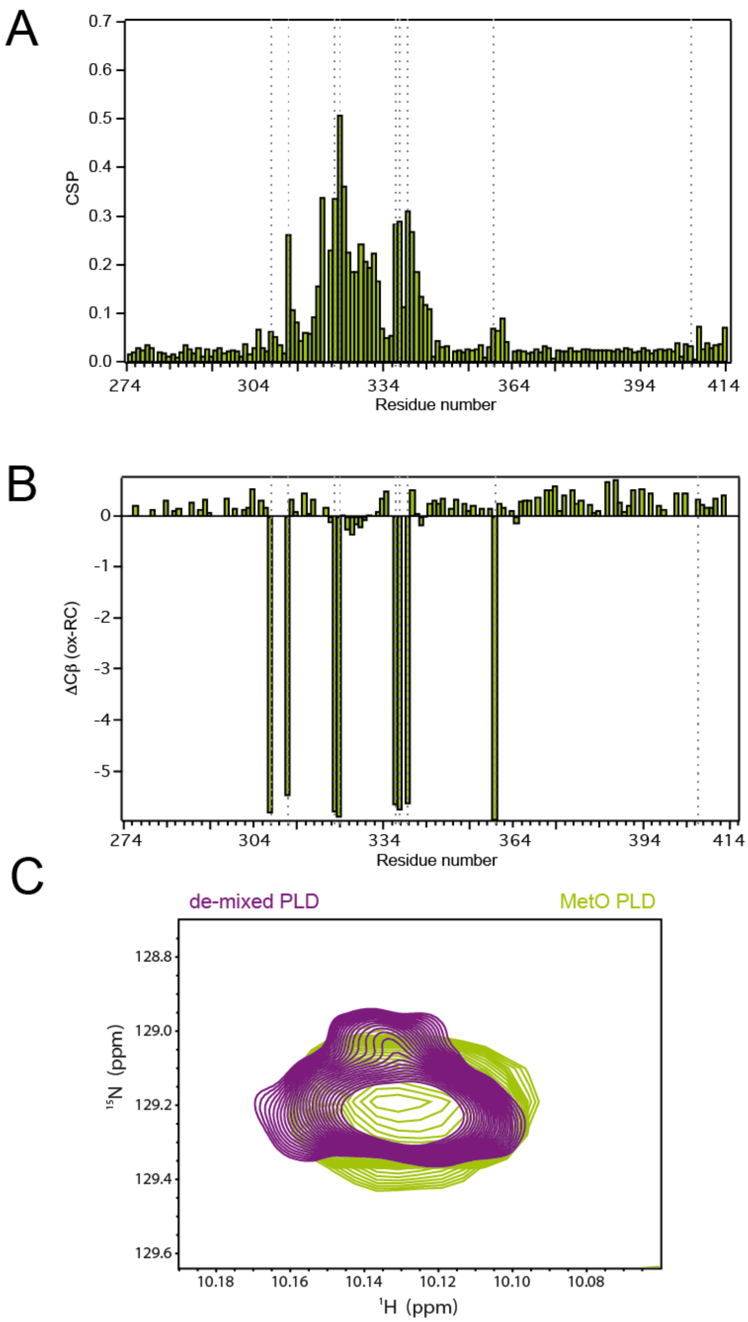

Supplementary Figure 5. MetO PLD is fully oxidized. A) Chemical shift perturbation plot (CSP) showing that methionine and nearby residues are significantly shifted in the ^{15}N-HSQC spectra upon methionine sulfoxidation. **B)** The chemical shifts for the Cβ atoms of the methionine residues shift significantly upon oxidation. The plot is obtained comparing the experimental Cβ chemical shifts for MetO PLD with the calculated shifts for PLD in a random coil (RC) conformation. Broken gray lines in the plots indicate the location of the methionine residues. Met405 assignments are absent. **C)** Detailed view of the overlaid ^{15}N-HSQC spectra for 300 μM unmodified PLD (purple) and MetO PLD (green) showing the Nε-Hε correlations for Trp sidechains. The lack of apparent shifts upon oxidation indicates that the Trp residues present in the PLD are not oxidized under the experimental conditions (**Supplementary Table 1**). Related to **Figures 1E-G.**

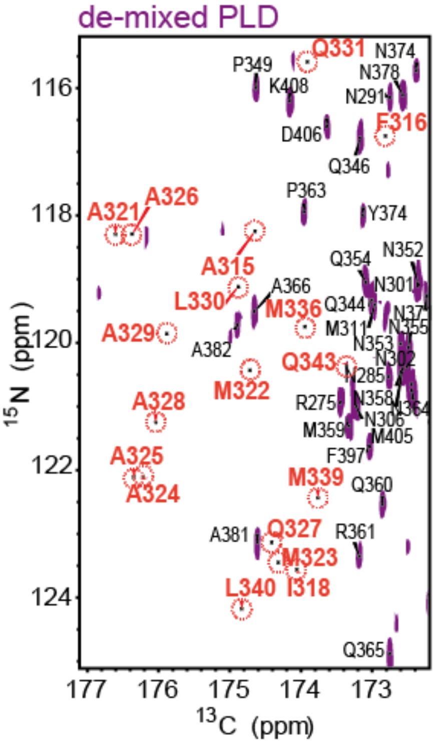

Supplementary Figure 6. Selected region of the CON spectrum of de-mixed PLD. The spectrum of a 300 μM sample of de-mixed PLD shows the absence of crosspeaks corresponding to the region 305-345. Assignments are shown in black, while the expected location of the missing peaks are labeled in red circles. Related to **Figure 1F.**

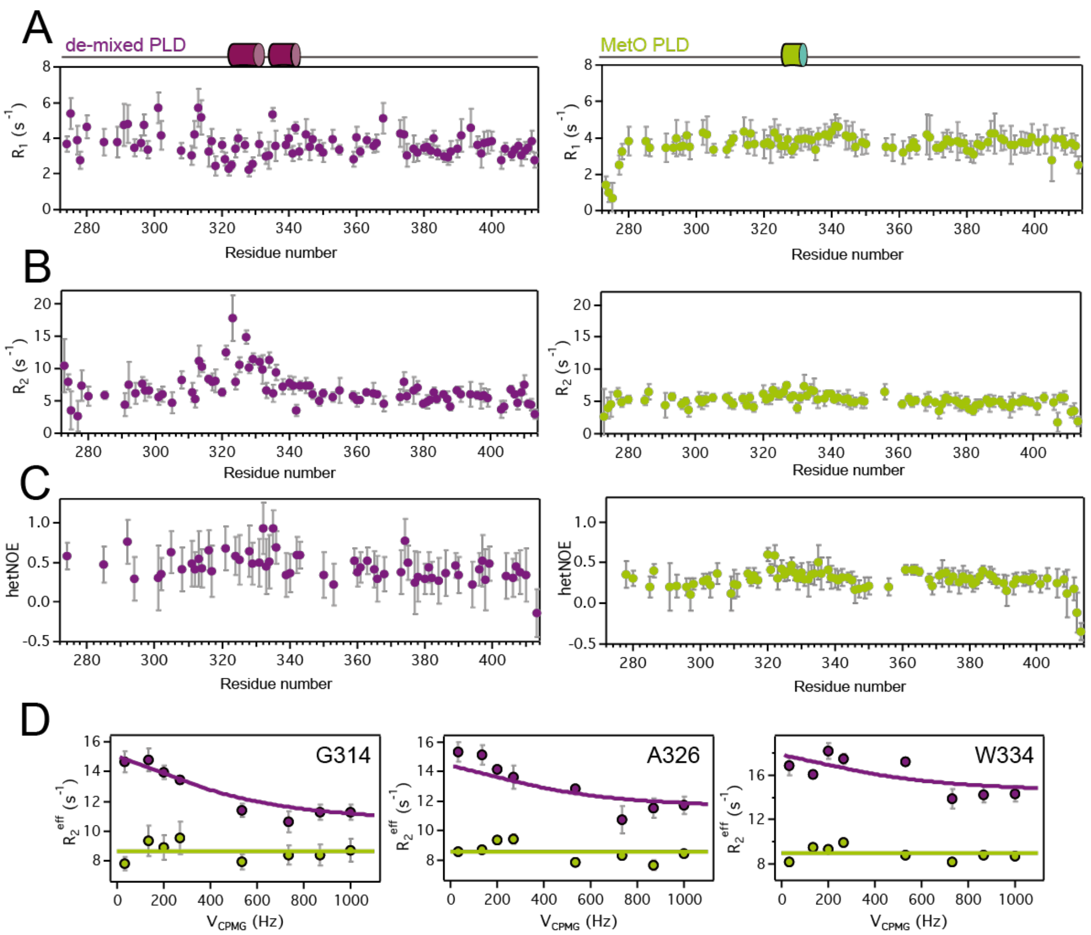

Supplementary Figure 7. Relaxation parameters for de-mixed PLD and MetO PLD. A-C) ^{15}N longitudinal (R_1, **A**), transverse (R_2, **B**) rate constants and heteronuclear (^{1}H)-^{15}N NOE (**C**) relaxation parameters obtained for 200 μM PLD (left, purple) and MetO PLD (right, green) at 800 MHz and 15 °C. Diagrams on top of the plots in (**A**) represent the α-helices formed in each protein. **D)** Representative ^{15}N CPMG relaxation dispersion profiles for 200 μM PLD (purple) and MetO PLD (green). Correlation time, order and exchange parameters are included in **Supplementary Table 2.** Related to **Figure 1F**.

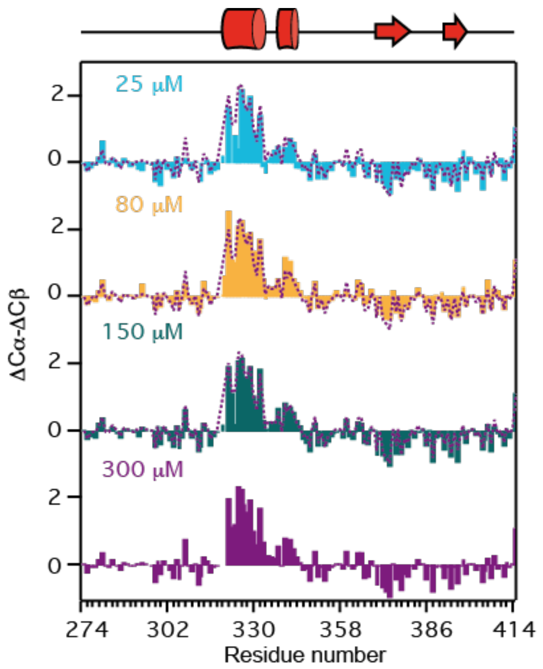

Supplementary Figure 8. Structural propensities of the PLD are concentration- and phase-independent. Secondary chemical shifts (calculated as ΔCα-ΔCβ) provide information on the structural propensities, since positive values indicate acquisition of α-helix, while negative values indicate acquisition of β-strands. Each plot shows the overlay with the data at 300 μM (broken purple line) for comparison. The schematic cartoon at the top highlights the double α-helix (represented as cylinders) and β-strands (arrows) formed in the PLD. Related to **Figure 1G.**

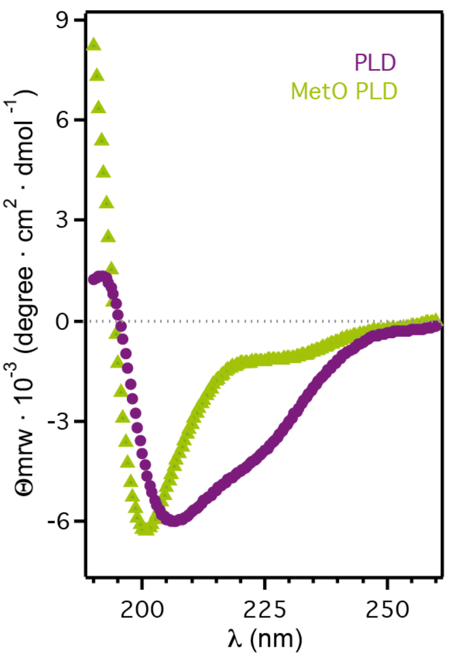

Supplementary Figure 9. MetO PLD undergoes a significant conformational rearrangement. Molar ellipticity plots for PLD (purple) and MetO PLD (green), showing a strong shift towards disordered conformations upon methionine sulfoxidation. 12 % α-helical content is estimated for the PLD following ($[\theta]_{222}/(-39500(1 - 2.57/n))$), where n is the number of total peptide bonds[1], while the NMR secondary chemical shifts indicate an overall 25% of α-helical content for the PLD (**Figure 1G**). The differences are mainly attributed to the different experimental temperatures (25 ^{o}C for CD and 15 ^{o}C for NMR).

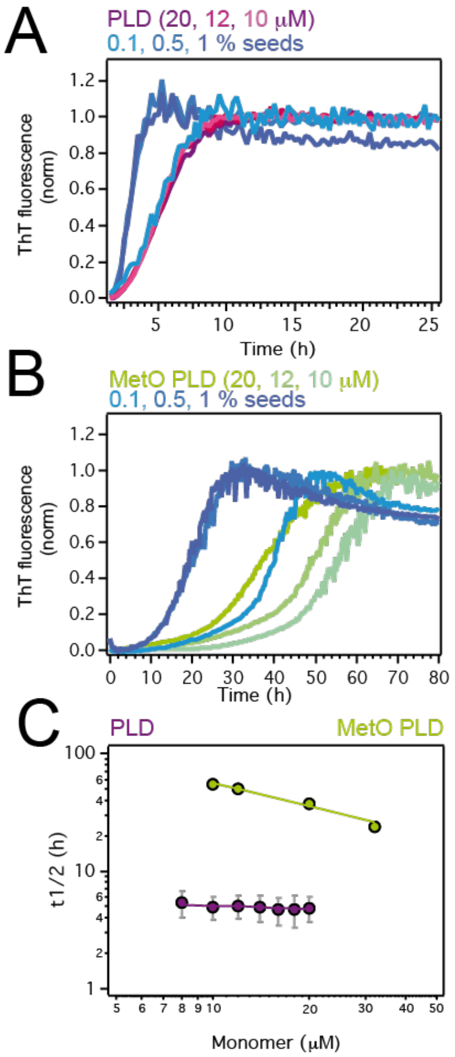

Supplementary Figure 10. Seeding accelerates PLD and MetO PLD aggregation kinetics. A) ThT aggregation kinetics for 20, 12 and 10 μM PLD and for 10 μM PLD in presence of increasing amounts of PLD seeds. **B)** ThT aggregation kinetics for 20, 12 and 10 μM MetO PLD and for 10 μM MetO PLD in presence of increasing amounts of MetO PLD seeds. Color codes are indicated. Errors are lower than 0.35 in (**A**) and 0.25 in (**B**) for normalized fluorescence intensity and error bars are not included for simplicity. **C)** Logarithmic plot of the time at which half of the monomer is converted to fibril, $t_{1/2}$, versus the initial monomer concentration (**Supplementary Table 3**). The scaling exponent largely differs between MetO PLD (-0.66) and PLD (-0.09), indicating that the aggregation is governed by distinct mechanisms[2]. Related to **Figure 2A.**

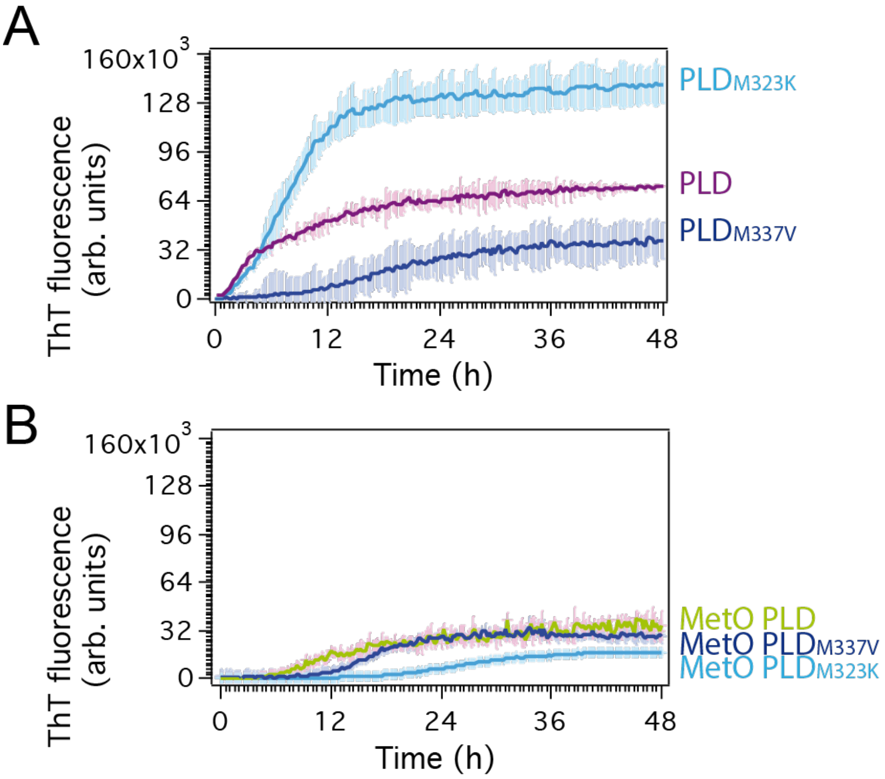

Supplementary Figure 11. ThT aggregation kinetics for PLD and disease-related mutants upon methionine sulfoxidation. While unmodified M323K shows a higher propensity to form fibrils than wild-type and M337V PLD, fibril formation is strongly suppressed for all MetO variants. Final protein concentration was 80 μM. Related to **Figure 2A.** n= 2 biologically independent experiments.

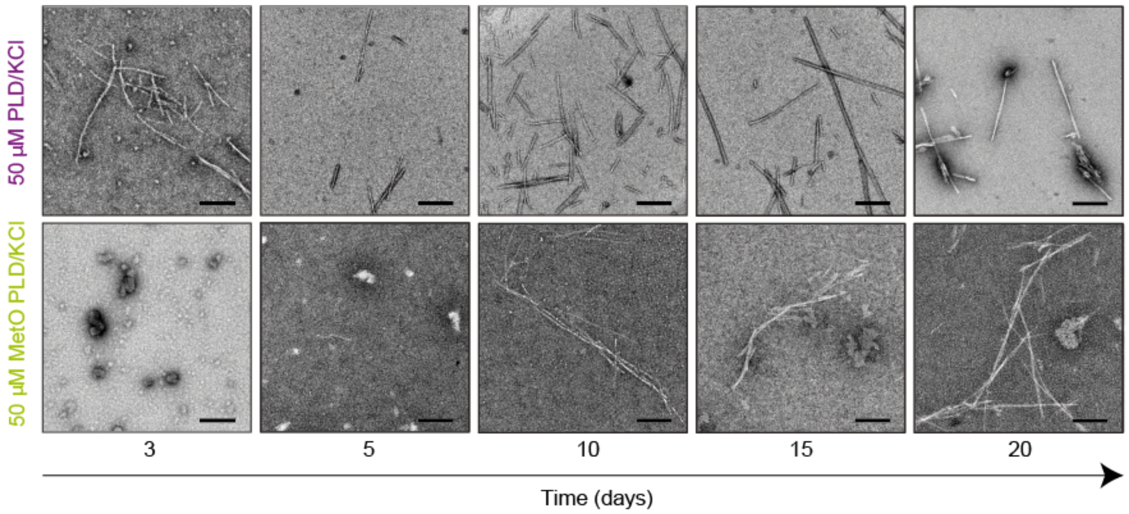

Supplementary Figure 12. Characterization of PLD and MetO PLD amyloid formation by TEM. Representative images obtained from 50 μM PLD (top) and 50 μM MetO PLD (bottom) samples aged at the indicated times. MetO PLD forms fibrils at a slower rate, and the fibrils formed are morphologically different. The scale bars represent 200 nm. Related to **Figures 2B-C.**

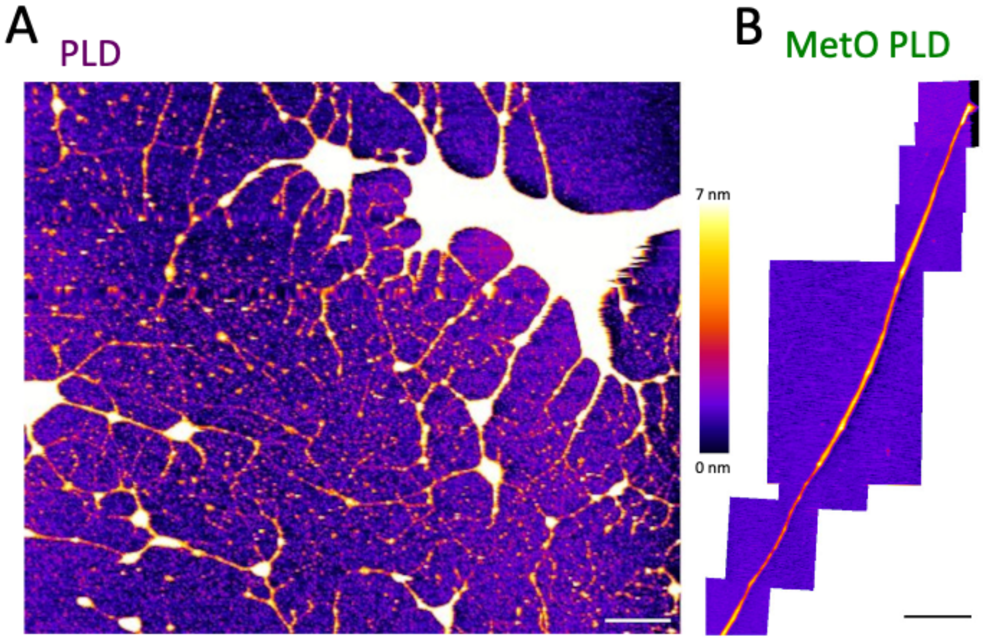

Supplementary Figure 13. Atomic force microscopy images of the amyloid fibrils formed by aged PLD and MetO PLD samples. 100 µM of PLD (**A**) and MetO PLD (**B**) incubated for 4 months were imaged on atomic-flat mica surfaces. The heat-color height scale corresponds to 7 nm, and scale bars correspond to 1 µm. Related to **Figure 2C.**

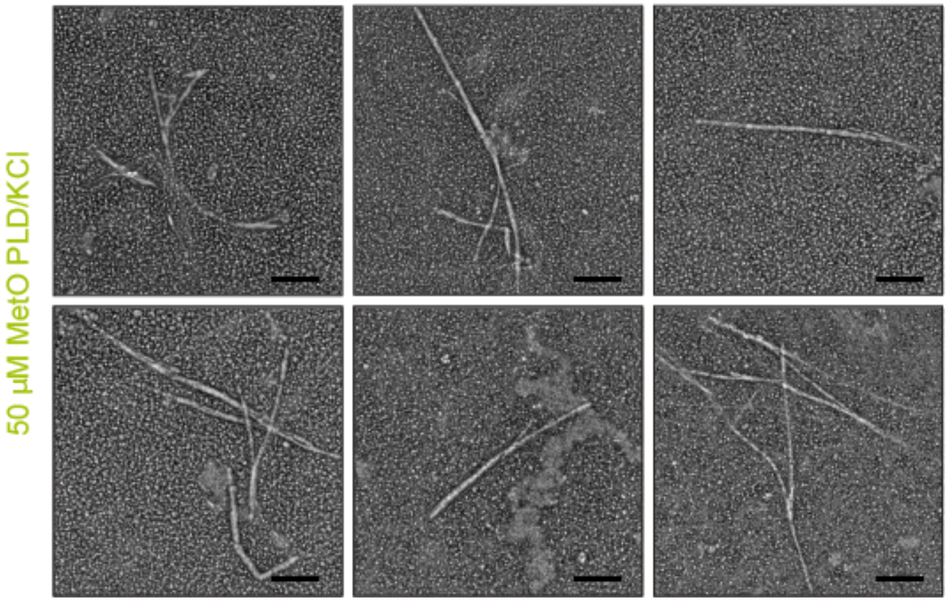

Supplementary Figure 14. Ultrastructure of aged MetO PLD fibrils. Representative TEM images of several aged fibrils formed by 50 μM MetO PLD. The morphology of the imaged fibrils resembles that of the long fibrils imaged by AFM (**Supplementary Figure 13B**). The scale bars correspond to 200 nm. Related to **Figure 2B.**

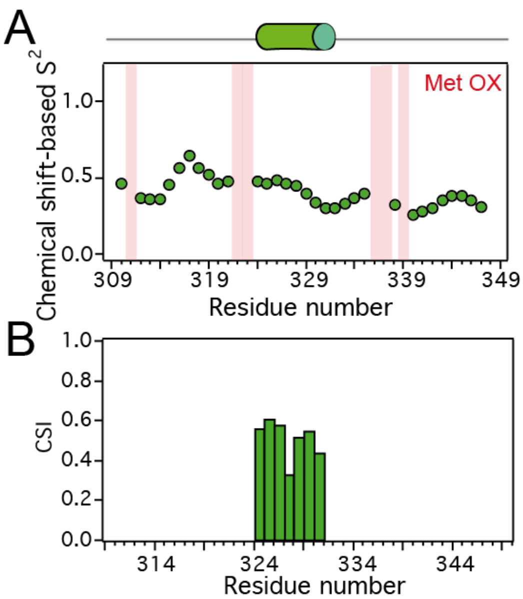

Supplementary Figure 15. NMR chemical shifts-derived parameters indicate that MetO PLD$_{309}$ is flexible. S^2 order parameters (**A**) and chemical shift index (**B**) obtained for MetO PLD$_{309}$ from TALOS-N. Light red bars in (**A**) indicate the location of Met residues. Cartoon representation on top locate the short α-helix formed in MetO PLD$_{309}$ (**Figure 3D**).

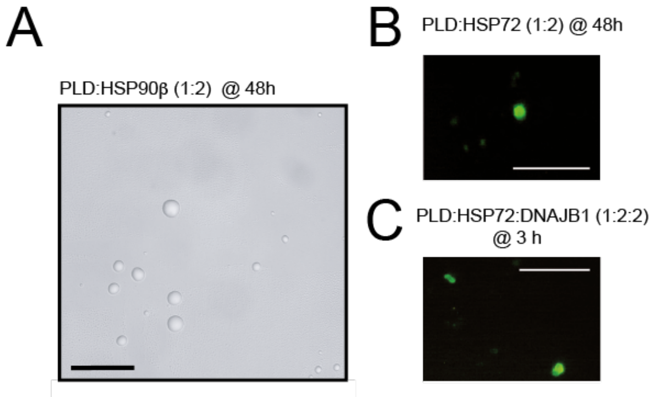

Supplementary Figure 16. Microscopy images for the condensates formed by the PLD in complex with chaperones and co-chaperones. A) DIC microscopy image revealing the condensates formed by the PLD in complex with HSP90β after 48 h of incubation at 25 °C. **B)** Fluorescence microscopy image of the condensates formed by the PLD labeled with Alexa Fluor 488 in complex with HSP72 after 48 h of incubation at 25 °C. **C)** Fluorescence microscopy image of the condensates formed by the PLD labeled with Alexa 488 in complex with HSP72:DNAJB1 after 3 h of incubation at 25 °C. Scale bars correspond to 25 μm. Samples contained 20 μM PLD and 150 mM KCl (turbidity conditions). Related to **Figures 4A-F.**

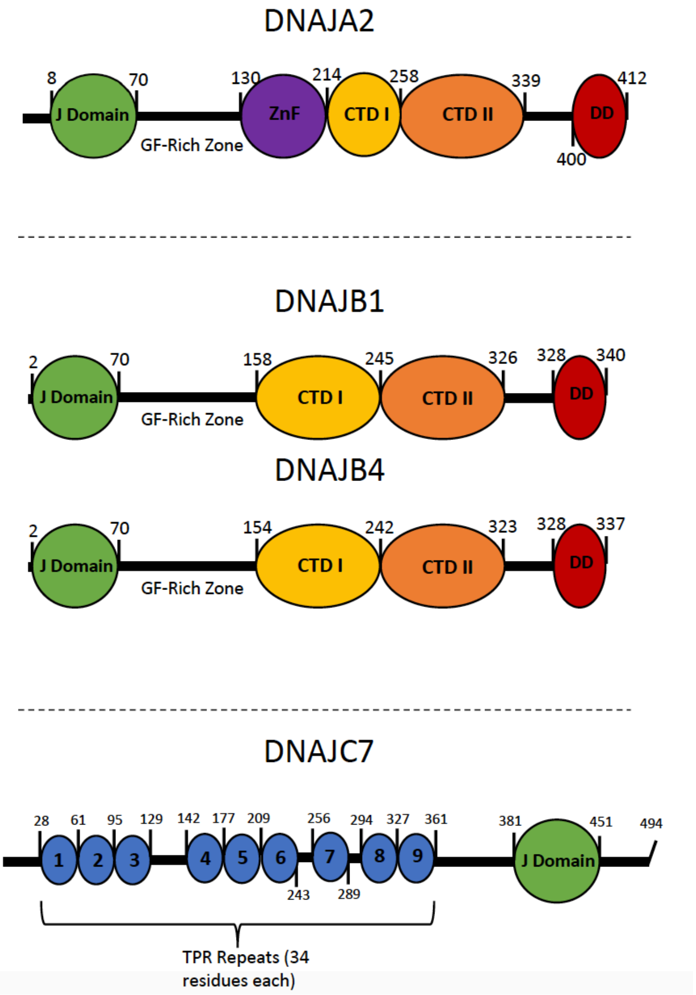

Supplementary Figure 17. Domain architecture for the JDPs characterized in this study.

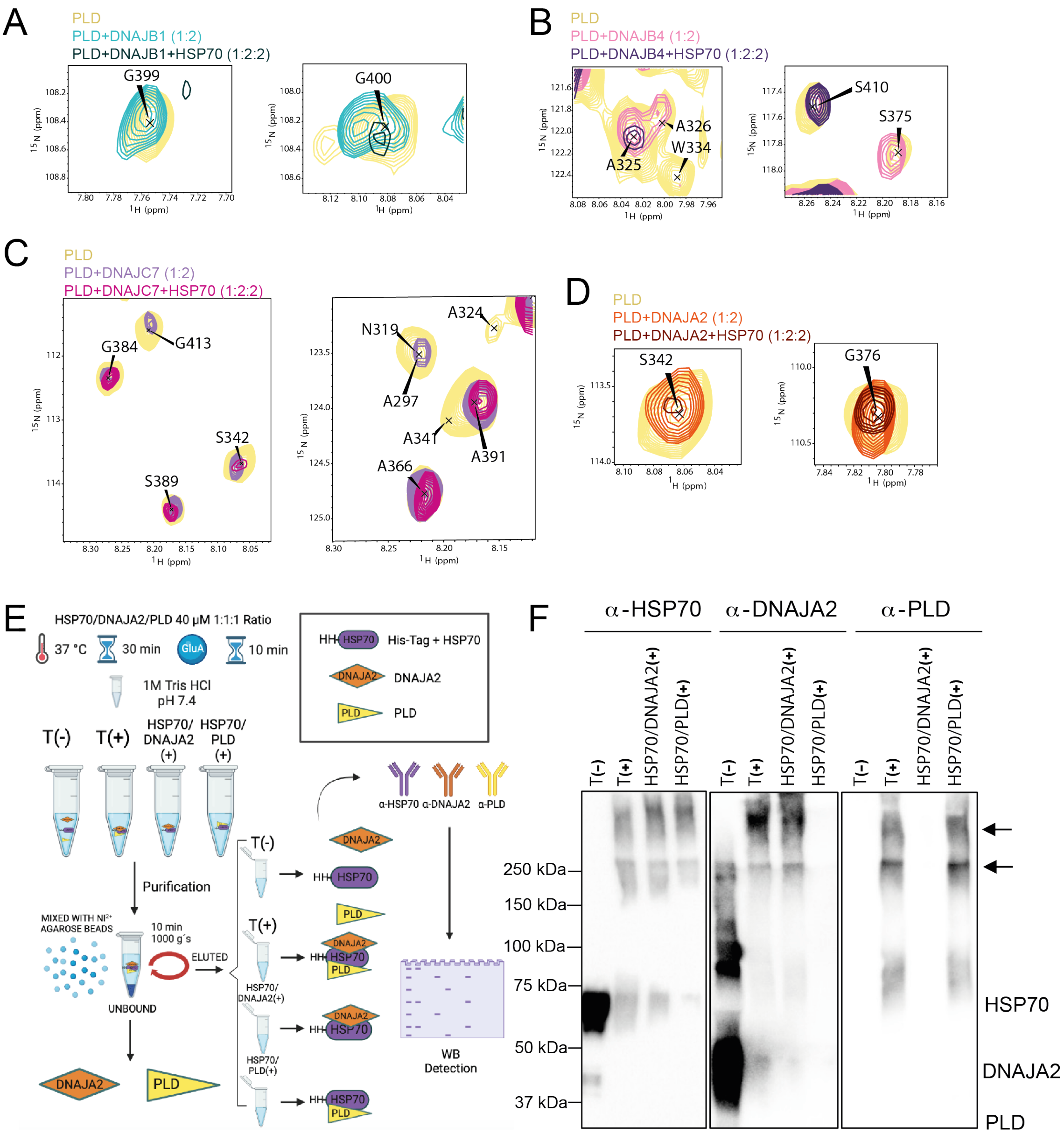

Supplementary Figure 18. The PLD is incorporated into the HSP70:JDPs complexes. A-D) Representative regions of the ^{15}N HSQC spectra for 25-35 μM PLD in the corresponding titration points. While binding to the JDPs promoted the disappearance of the signals from the PLD interacting residues by slow exchange, addition of HSP70 to the preformed PLD:JDP complexes induced further signal decay in specific residues. This serves as an indication of ternary complex formation. **E)** Schematic representation (created with BioRender.com) of the chemical crosslinking experiment to validate the formation of the PLD:HSP70:JDP ternary complex. DNAJA2 was used as a representative JDP. Samples contained 40 μM PLD and the corresponding molar ratios. (-) and (+) symbols represent absence and presence of crosslinker, respectively. Only HSP70 contained a Histag, facilitating its pull-down by Ni^{2+}-loaded agarose. **F)** Immunoblotting results for the crosslinking of the PLD:HSP70:DNAJA2 ternary complex. Several high molecular weight bands (marked with arrows) appear upon crosslinking and are revealed by the three antibodies, confirming ternary complex formation. HSP70 is 72 kDa, DNAJA2 45 kDa and the PLD 13.8 kDa. Related to **Figure 4G.**

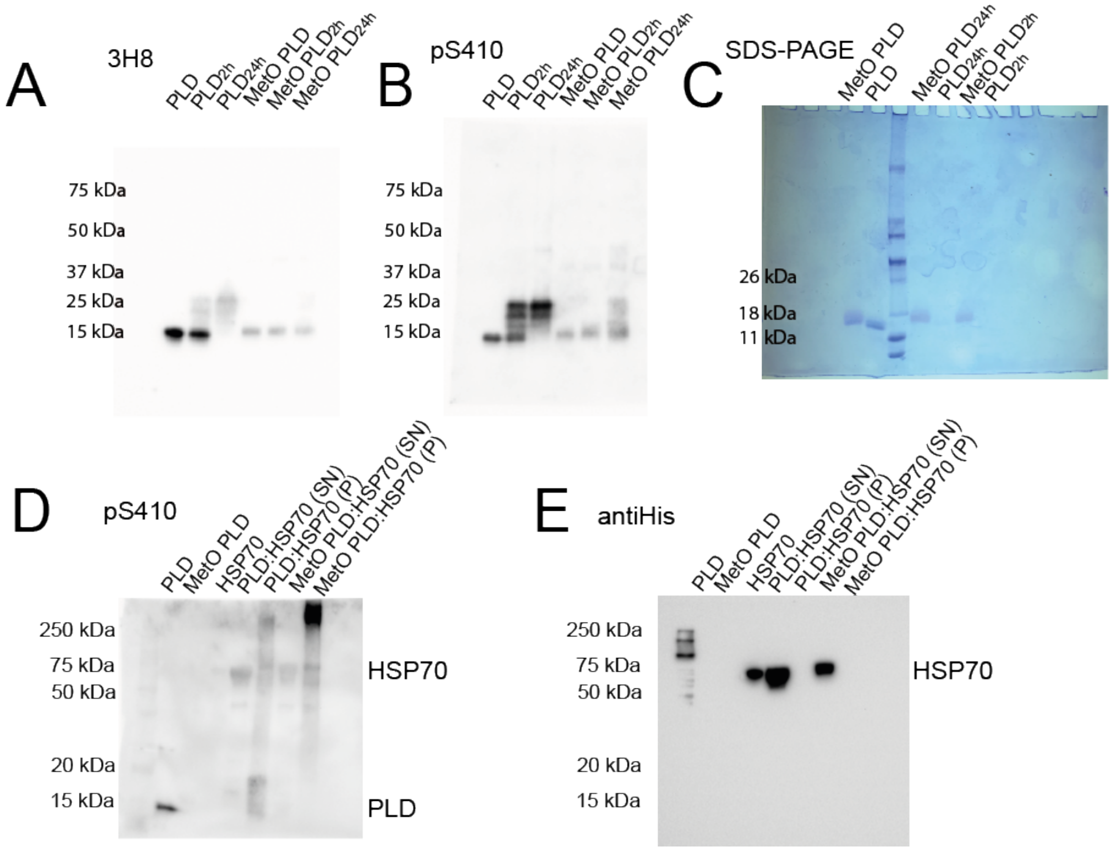

Supplementary Figure 19. Immunoblotting confirms the aggregation of phosphorylated PLD. A-C) Uncropped images of the western blot immunoblotting results using 3H8 antibody (**A**), pS410 (**B**) and SDS-PAGE (**C**), related to **Figure 5A**. CK1δ incubation times are indicated. **D-E)** Western blotting of the CK1δ phosphorylated samples after the NMR experiments (related to **Figure 5D**). Phosphorylation by CK1δ promoted significant oligomerization, observed by the appearance of high molecular weight bands in the phosphorylated samples. NMR samples were clarified at 20,000 g prior to the western blotting, and fractions separated in supernatant (SN) and pellet (P). PLD, MetO PLD and HSP70 samples were included as controls. The His-tag present in HSP70 was used for detection in western blotting (**E**). Despite the unspecific labeling of unmodified PLD, pS410 antibody reveals phosphoPLD aggregates. MetO PLD large aggregates are also reactive against pS410 antibody. HSP70 remained in the soluble fractions.

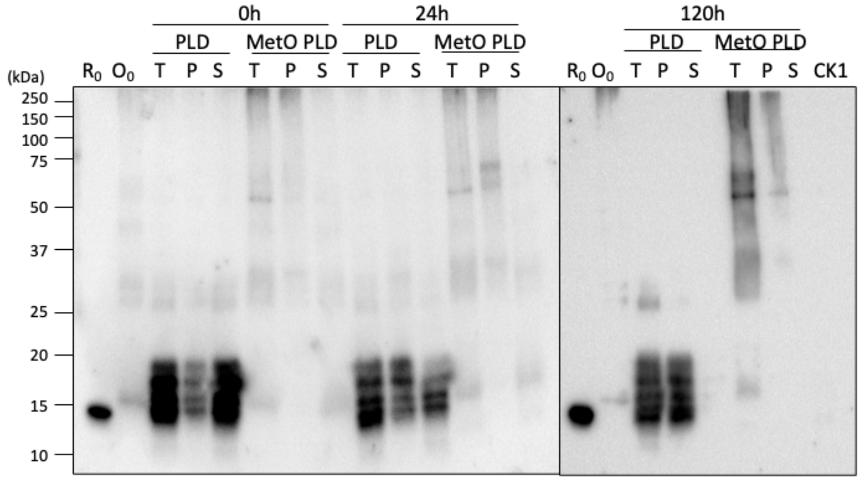

Supplementary Figure 20. CK1δ phosphorylates PLD monomer and MetO PLD fibril. 20 μM PLD and MetO PLD were incubated at 37 °C and 0.4 μM CK1δ was added at the indicated time points (0, 24 and 120 h) and left for 1 hour at 37 °C. The immunoblot was developed using anti pS410 antibody. R_0 and O_0 (for reduced and oxidized PLD without CK1δ, respectively) samples were incubated in the absence of CK1δ, *T* indicates total fraction, *P* indicates pellet and *S* soluble fractions. Phosphorylated monomers are resolved as discrete bands of 15-19 kDa, whereas phosphorylated aggregates run as a 25-250 kDa smear. Related to **Figure 5.**

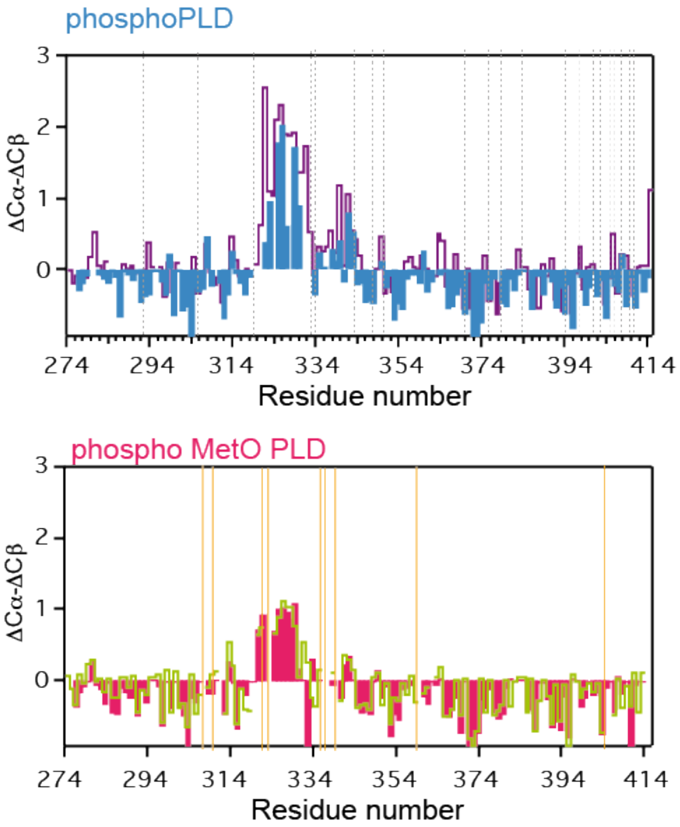

Supplementary Figure 21. Secondary chemical shifts in presence of CK1δ. Secondary chemical shifts for 40 μM phosphoPLD (top, light blue) and 40 μM phospho MetO PLD (bottom, magenta). For comparison, data for 80 μM PLD (purple line, top) and 300 μM MetO PLD (green line, bottom) are included in the plots. Gray broken lines (top) locate Ser residues, while golden lines (bottom) locate Met residues on the plots. Related to **Figure 5.**

Residue	Post-translational Modification	Percentage
N285	Deamidation	99.7%
Q286	Deamidation	99.5%
N291	Deamidation	100%
N301	Deamidation	100%
N306	Deamidation	100%
**M307**	Oxidation	100%
**M311**	Oxidation	100%
N312	Deamidation	100%
N319	Deamidation	98.9%
**M322**	Oxidation	75%
**M323**	Oxidation	99.5%
Q331	Deamidation	98.7%
**M336**	Oxidation	100%
**M337**	Oxidation	100%
**M339**	Oxidation	100%
Q346	Deamidation	97%
N352	Deamidation	99.7%
N353	Deamidation	95.4%
N358	Deamidation	100%
**M359**	Oxidation	99.6%
N372	Deamidation	98.4%
N378	Deamidation	97,9%
N390	Deamidation	100%
N398	Deamidation	100%
**M405**	Oxidation	100%

Supplementary Table 1. List of modifications detected by mass spectrometry in MetO PLD. Met residues are highlighted in red. The C-terminal Met residue (M414) was not detected. Considering the sequence properties of the PLD, long peptide digestions were necessary at neutral/alkaline pH, which can lead to artificial deamidation[3]. In addition, no modifications in the N/Q residues present above were observed in the NMR spectra. Therefore, we conclude that the deamidation modifications result from sample preparation.

	T_1 (s)	T_2 (s)	(^{1}H)-^{15}N NOE	S^2	τ_C (ns)	k_{ex} (s^{-1})	p_B
PLD	0.30 +/- 0.07	0.13 +/- 0.05	0.56 +/- 0.15	0.51 +/- 0.17	3.3	>2000	4 ± 0.5 %
MetO PLD	0.26 +/- 0.02	0.17 +/- 0.02	0.38 +/- 0.09	0.40 +/- 0.13	1.5	n.d.	n.d.

Supplementary Table 2. Correlation time, order and exchange parameters for the region 321-343 of PLD and MetO PLD. S^2 were derived from the chemical shifts[4], the overall correlation time (τ_C) from the ratio of the mean values of T_1 and T_2[5], and k_{ex} and p_B from ^{15}N CPMG relaxation dispersion data. Increased S^2 and τ_C for the PLD indicate that the protein is less flexible and assembles into larger species, respectively, while MetO PLD is more flexible and remains disperse. MetO PLD showed no conformational exchange in the μs-ms time domain. Data are related specifically to the structured region of the PLD (comprising residues 321-343). n. d: not detected.

% Seeds	$t_{1/2}$ (h)	
	PLD	MetO PLD
0	4.87 ± 0.37	54.88 ± 1.60
0.1	4.99 ± 0.31	38.26 ± 0.20
0.5	2.88 ± 0.06	18.70 ± 0.28
1	2.86 ± 0.15	18.02 ± 0.22

Supplementary Table 3. Dependence of the time at which half of the monomer is converted to fibril, $t_{1/2}$, on the amount of seeds present for PLD and MetO PLD.

NOE distance constraints:<br>Intraresidual<br>Dihedral angle restraints<br>Stereospecific ^{1}H assignments<br>Total<br>Maximal violation (Å)	 56<br>24<br>0<br>80<br>0.22
CYANA target energy function (Å^2)	0.05 ± 0.04
RMSD (to mean coordinates):<br>Heavy backbone (Å) (segment 324-332)<br>All heavy side chain atoms (Å)	 0.41<br>1.38
Procheck Ramachandran plot statistics:<br>Most favored regions (%)<br>Additionally allowed favored regions (%)	 73.5<br>26.5

Supplementary Table 4. Structure Calculation Statistics for MetO PLD$_{309}$.

References

1. Sommese RF, Sivaramakrishnan S, Baldwin RL, Spudich JA. Helicity of short E-R/K peptides. *Protein Sci* **19**, 2001-2005 (2010).

2. Thacker D, Sanagavarapu K, Frohm B, Meisl G, Knowles TPJ, Linse S. The role of fibril structure and surface hydrophobicity in secondary nucleation of amyloid fibrils. *Proc Natl Acad Sci U S A* **117**, 25272-25283 (2020).

3. Krokhin OV, Antonovici M, Ens W, Wilkins JA, Standing KG. Deamidation of -Asn-Gly- sequences during sample preparation for proteomics: Consequences for MALDI and HPLC-MALDI analysis. *Anal Chem* **78**, 6645-6650 (2006).

4. Berjanskii MV, Wishart DS. A simple method to predict protein flexibility using secondary chemical shifts. *J Am Chem Soc* **127**, 14970-14971 (2005).

5. Kay LE, Torchia DA, Bax A. Backbone dynamics of proteins as studied by ^{15}N inverse detected heteronuclear NMR spectroscopy: application to staphylococcal nuclease. *Biochemistry* **28**, 8972-8979 (1989).
